# Supplementary material for: MetaRibo-Seq measures translation in microbiomes
Source: Nat Commun. 2020 Jun 29;11:3268. doi: 10.1038/s41467-020-17081-z (PMC7324362; doi:10.1038/s41467-020-17081-z)
Supplement: Supplementary file 10 — Supplementary Data 7 [file 41467_2020_17081_MOESM10_ESM.zip › File2/Confidence_High_Taxonomy/155801_out.krona.html]

Javascript must be enabled to view this page.

members
magnitude
magnitudeUnassigned
count
unassigned
taxon
rank

155801\_out

41

superkingdom
41
2

phylum
976
41

class
200643
41

171549
41
order

815
41
family

816
41
genus

371601
1
species

SRS062427\_contig\_number\_9089

821
38
species

SRS011239\_contig\_number\_12901SRS011302\_contig\_number\_30356SRS011405\_contig\_number\_17116SRS012902\_contig\_number\_319SRS014287\_contig\_number\_16339SRS014459\_contig\_number\_30663SRS015065\_contig\_number\_30501SRS015486\_contig\_number\_1001SRS015579\_contig\_number\_18036SRS016203\_contig\_number\_9063SRS017191\_contig\_number\_11528SRS017375\_contig\_number\_270SRS020622\_contig\_number\_3847SRS022137\_contig\_number\_12376SRS023829\_contig\_number\_39SRS045004\_contig\_number\_11095SRS045645\_contig\_number\_2077SRS055017\_contig\_number\_27573SRS055982\_contig\_number\_12700SRS063518\_contig\_number\_38446SRS075398\_contig\_number\_929SRS078242\_contig\_number\_20165SRS101376\_contig\_number\_7083SRS101433\_contig\_number\_14672SRS101441\_contig\_number\_contig-100\_6090.6090SRS1041036\_contig\_number\_4088SRS1041039\_contig\_number\_10252SRS1041144\_contig\_number\_8210SRS1041157\_contig\_number\_5584SRS1041164\_contig\_number\_796SRS104400\_contig\_number\_24568SRS104912\_contig\_number\_15548SRS1054928\_contig\_number\_2866SRS1055083\_contig\_number\_8266SRS143070\_contig\_number\_10144SRS146812\_contig\_number\_63780SRS147271\_contig\_number\_41814SRS971275\_contig\_number\_47

2
29523
species

SRS045244\_contig\_number\_2627SRS1041137\_contig\_number\_28734
